# Ozone Application Suppressed the Blue Mold Development and Maintained the Main Active Ingredients Content of Postharvest Fresh *Codonopsis pilosula* during Storage

**DOI:** 10.3390/jof10030163

**Published:** 2024-02-20

**Authors:** Jiangyang Chen, Zhiguang Liu, Qili Liu, Dan Zhang, Huali Xue, Suqin Shang, Yang Bi

**Affiliations:** 1 College of Science, Gansu Agricultural University, Lanzhou 730070, China; jiangyangchen@sinopeg.com (J.C.); liuzg@st.gsau.edu.cn (Z.L.); zhangdan@gsau.edu.cn (D.Z.); 2 College of Food Science and Engineering, Gansu Agricultural University, Lanzhou 730070, China; lql744607@163.com (Q.L.); biyang@gsau.edu.cn (Y.B.); 3 College of Plant Protection, Gansu Agricultural University, Lanzhou 730070, China

**Keywords:** *Penicillium expansum*, *Codonopsis pilosula*, ozone, main active ingredients, patulin

## Abstract

*Penicillium expansum* is the predominant causal agent causing blue mold in postharvest fresh *Codonopsis pilosula* during storage. The pathogen reduces the yield and affects the quality of *C. pilosula* and even generates patulin, threatening human health. In this study, postharvest fresh, healthy *C. pilosula* was sprayed with *P. expansum*, and the control effect of ozone on postharvest diseases of *C. pilosula* was studied, and the effect of ozone on the contents in the main active ingredients of *C. pilosula* was compared; finally, the effect of ozone on reactive oxygen species (ROS) metabolism in *C. pilosula* was analyzed. The results showed that 2 mg L^−1^ ozone application significantly inhibited the occurrence of postharvest blue mold caused by *P. expansum*, reduced weight loss rate, controlled the accumulation of patulin and maintained the contents of the main active components in *C. pilosula*. The study will provide a theoretical basis for ozone treatment to control the occurrence of postharvest diseases of *C. pilosula.*

## 1. Introduction

*Codonopsis pilosula* is a perennial herb of Campanulaceae [1], whose main active ingredients are lobetyolin, codonopsine, volatile oil, flavonoids, phytol and polysaccharides [2]. Phenylpropanoid glycosides are a major functional component isolated from the dried roots of *C. pilosula*. These compounds have rich medicinal value [3], which can enhance immunity [4], improve microcirculation [5], enhance hematopoietic function [6], and have obvious improvement effects on diabetes, liver protection and base repair [7].

Gansu Province is the dominant producer of *C. pilosula* in China, with a planting area of 50,000 hm^2^ and a yield of 558,000 tons [8]. However, with the continuous expansion of *C. pilosula* planting area, the disease of *C. pilosula* caused by pathogenic fungi has gradually become more obvious, which seriously affects the yield and quality of *C. pilosula*. The infection caused by pathogenic fungi not only leads to the loss of Chinese medicinal materials, but also causes mycotoxin contamination in Chinese medicinal materials, which seriously reduces the quality of Chinese medicinal materials and impairs their therapeutic effect, and even endangers the health of patients.

*Penicillium expansum* is a predominant causal agent, which was isolated from the blue mold of fresh *C. pilosula* during storage by our research team [9]. The infection of *P. expansum* not only leads to the occurrence of postharvest blue mold in *C. pilosula*, but also produces patulin with carcinogenic, teratogenic and mutagenic effects [10]. Many countries have stipulated the maximum limit of patulin in food. The European Union (EU) and the US Food and Drug Administration (USFDA) have stipulated that the maximum limit of patulin in fruit juice drinks is 50 μg/L [11]. However, there is no report on the stipulated limit of patulin in Chinese medicinal materials. As a traditional Chinese medicine for both medicine and food, the quality of *C. pilosula* is closely related to the health of patients. At present, the wide application of chemical synthetic fungicides not only leads to the resistance of pathogens, but also raises the problems of chemical residues, environmental pollution and the safety of medicinal materials.

As an excellent strong oxidant, the most significant feature of ozone is that it can be decomposed into O_2_ and singlet oxygen atom without any residue after application; therefore, ozone is called non-polluting disinfectant [12]. Ozone not only has a strong ability to kill various fungi, but can also reduce the accumulation of mycotoxins and degrade pesticide residue to a certain extent [13]. Ozone is widely applied in the prevention and control of postharvest diseases of fruits and vegetables [14]. The mechanism of ozone involved in postharvest disease control is that, on the one hand, ozone can directly affect the growth and development of the pathogenic fungus by destroying the cell structure; on the other hand, ozone treatment can induce resistance to the pathogen by activating the active oxygen metabolism (ROS) of the host plant to increase the antioxidant enzymatic activity, then avoid excessive accumulation of reactive oxygen species. Evidence has shown that ozone treatment controlled postharvest diseases of kiwifruit by inhibiting the growth of *Botrytis cinerea* and *P. expansum* and increased the activity of defense-related enzymes [15]. However, the effect of ozone treatment on the control of blue mold, patulin accumulation and the main active ingredient contents of fresh *C. pilosula*, as well as the possible mechanism of action, are rarely reported.

In this study, the effects of ozone treatment on the disease development, weight loss rate, and patulin accumulation of *C. pilosula* were firstly investigated by spraying inoculation *P. expansum* spore suspension. The influence of ozone exposure on the changes of the main active ingredients in *C. pilosula* after *P. expansum* inoculation was determined. Finally, the possible action mechanism was analyzed.

## 2. Materials and Methods

### 2.1. Materials

Fresh *C. pilosula* samples were purchased from *C. pilosula* planting base in Min County (location 35° N and 104° E), Gansu Province of China. Roots with a similar size and without obvious pest or mechanical damage were collected in November 2021, taken to the Chemical Biology Laboratory in Gansu Agricultural University and stored at room temperature for further experiment.

*P. expansum* was isolated and purified from the tissue of *C. pilosula* with typical symptoms of the blue mold of *C. pilosula* by our research team and identified by morphology and molecular biology [16], and was stored at 4 °C for later use.

### 2.2. Methods

#### 2.2.1. Preparation of Spore Suspension

*P. expansum* spore suspension was prepared based on the method of Luo et al. [15]. 10 mL sterile water (containing 0.01 mL Tween 80) was added to *P. expansum* cultured for 7 days. The concentration of the filtered spore suspension was adjusted to 1 × 10^6^ spores mL^−1^.

#### 2.2.2. Ozone Treatment Method

The healthy roots of *C. pilosula* were disinfected with 1% sodium hypochlorite for 5 min, washed with sterile distilled water to remove sodium hypochlorite remaining on the surface of the roots, and dried naturally in the air. The above prepared 1 × 10^6^ spores mL^−1^ *P. expansum* spore suspension was evenly sprayed on the surface of *C. pilosula* (spray volume of 3 mL). After 2 h of inoculation, ozone treatment was performed by ozone generator (OSAN, Aoshan Huanbao Technology Industry Co., Ltd., Dalian, China). In our previous study, we did a preliminary experiment on the ozone concentration screening. The results showed that 2 mg L^−1^ of the ozone concentration had the optimal inhibition effect on the disease development [17]. Therefore, the *C. pilosula* inoculated with *P. expansum* was treated with 2 mg L^−1^ ozone gas in a tightly sealed air bottle (diameter 20 cm, height 25 cm) for 0, 1 and 2 h, respectively.

#### 2.2.3. Analysis of Incidence of the Blue Mold and Patulin Accumulation of *C. pilosula* during Different Storage Periods

*C. pilosula* inoculated with *P. expansum* was treated with ozone at different times, and then kept at 15 °C and 50% RH. The incidence was observed every 7 days and the disease index (formula (1)) and disease incidence (formula (2)) were calculated [18]; the disease classification standards are shown in Table S1 (Supplementary Materials, Table S1).

Disease index = [(Diseased plants at each level × Number of plants at that level)/(Total number of plants × Highest disease level)] × 100%,(1)

Disease incidence= (Diseased plants investigated/Total plants investigated) × 100%,(2)

The *C. pilosula* inoculated with *P. expansum* but not treated with ozone was used as a control. The samples were collected every 7 days for patulin analysis. Patulin accumulation was determined based on the method by Lv [19].

#### 2.2.4. Determination of Weight Loss Rate of *C. pilosula* during Different Storage Periods

The *C. pilosula* inoculated with *P. expansum* was treated with ozone for 0, 1 and 2 h, respectively, then packaged with sterile bags and kept at room temperature (15 °C, 50% RH). The weight of the treated *C. pilosula* was weighed by analytical balance every 7 days. The weight loss rate was calculated based on the following formula (3).

Weight loss rate = [Initial weight(W1) − Storage time node weight(W2)]/[Initial weight(W1)] × 100%,(3)

#### 2.2.5. Analysis of Main Active Ingredients in *C. pilosula* Inoculated with *P. expansum* during Different Storage Periods

The single standard solutions of main active ingredients were prepared as follows: 1 mg lobetyolin, 0.5 mg syringin, 0.5 mg lobetyolin I, 0.5 mg atractylenolide I, 0.5 mg atractylenolide II and 0.5 mg atractylenolide III were dissolved separately in 5 mL methanol to obtain the six single standard stock solutions. The mixed standard solution was done as follows: the above-mentioned single standard solutions were accurately measured and transferred into the 10 mL volumetric flask, and methanol was added to obtain the mixed standard solution of the above six main active ingredients.

Chromatographic conditions: (Supplementary Materials, Table S2).

The preparation of *C. pilosula* samples was carried out according to the above Section 2.2.3 method. A 1.0 g sample of powder was accurately weighed, transferred into a centrifuge tube to extract with 25 mL methanol, vortexed in a vortex mixer for 3 min, then ultrasonically extracted at 20 °C (40 kHz, 150 W) for 30 min. The extract was obtained and centrifuged at 4 °C, 5000× *g* for 5 min. The supernatant was evaporated and concentrated to dryness at 45 °C. The residue was redissolved with methanol and transferred to a 5 mL volumetric flask. Methanol was added to a constant volume of 5 mL and, finally, filtered through a 0.45 µm organic microporous membrane to further HPLC analysis.

#### 2.2.6. Analysis of ROS Metabolism in *C. pilosula* Inoculated with *P. expansum* during Different Storage Periods

The production of superoxide anion (O_2_^−.^) and the content of hydrogen peroxide (H_2_O_2_) were determined according to the method of Bao et al. [20]. The production of O_2_^−.^ content was expressed as nmol/g FW (fresh weight). H_2_O_2_ content was expressed as mmol/g FW.

NADPH oxidase (NOX) activity was determined using NADPH oxidase kit (Suzhou Keming Biotechnology Co., Ltd., Suzhou, China). NOX activity was expressed as U/g FW. Superoxide dismutase (SOD) activity was determined using superoxide dismutase kit (Suzhou Keming Biotechnology Co., Ltd. China Suzhou Limited). SOD activity was expressed as U/g FW. Catalase (CAT) activity was determined referring to the Fan et al. [21] method. CAT activity was expressed as U/min/g FW. Peroxidase (POD) activity was determined referring to the method of Venisse et al. [22]. POD activity was expressed as ΔOD470/min/g FW.

#### 2.2.7. Determination of Cell Membrane Permeability

Cell membrane permeability was measured and calculated according to the method of Chen et al. [23].

#### 2.2.8. Determination of Malondialdehyde Content

Malondialdehyde (MDA) content was determined according to the method of Li et al. [24].

#### 2.2.9. Data Statistics and Analysis

The experimental data were expressed as mean ± standard deviation. SPSS 26.0 statistical analysis software was used for data processing. The significance of the difference was expressed at the *p* < 0.05 level, and the software Origin 2022 was used for mapping.

## 3. Results

### 3.1. Ozone Treatment Controlled the Development of the Blue Mold of C. pilosula 

**Figure 1:**
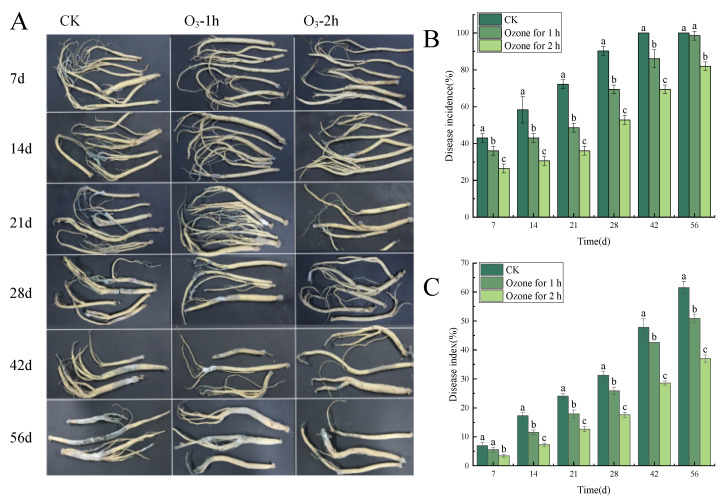
Effects of ozone treatment on the disease incidence and disease index of the blue mold of *C. pilosula* during different storage periods. (**A**): Pictures of disease symptoms of *C. pilosula* samples during different storage periods; (**B**): Disease incidence; (**C**): Disease index. Bars indicate standard error. Different letters indicate significant differences (*p* < 0.05).

Ozone treatment significantly (*p* < 0.05) inhibited the occurrence of the blue mold of in *C. pilosula* during the postharvest storage period (Figure 1A). With the extension of storage time, the disease incidence and disease index showed an increasing trend. For example, on the 7th day of storage after treatment, the control group had obvious root disease with 1~2 mm granular mold spots on the surface, while the same symptoms were observed on the 14th day of storage for the ozone treatment group; that is to say, compared with the control group, the development of the disease was delayed by 7 days. On the 42th day of storage, the disease incidence of the control group increased rapidly; almost all samples were infected and some sample roots were decayed or worse, with soft rot and obvious mildew on the surface of roots. However, compared with the control group, the disease incidence of the ozone treatment group was significantly lower than that of the control group, and the longer the ozone treatment time, the better the inhibition of blue mold of *C. pilosula*. As shown in Figure 1B, on the 42nd day, the disease incidence of the ozone treatment 1 h group and the ozone treatment 2 h group was 13.9% and 30.5% lower than those of the control group, respectively. On the 56th day of storage, the control group displayed severe symptoms of disease, the incidence of which was 21.3% higher than that of the ozone treatment 2 h group (Figure 1C). In brief, ozone treatment significantly inhibited the expansion of the blue mold of *C. pilosula* caused by *P. expansum* and delayed the disease incidence of *C. pilosula*, and the longer the ozone treatment time, the better the inhibition effect that was obtained.

### 3.2. Ozone Treatment Suppressed the Weight Loss Rate of C. pilosula Inoculated with P. expansum

**Figure 2:**
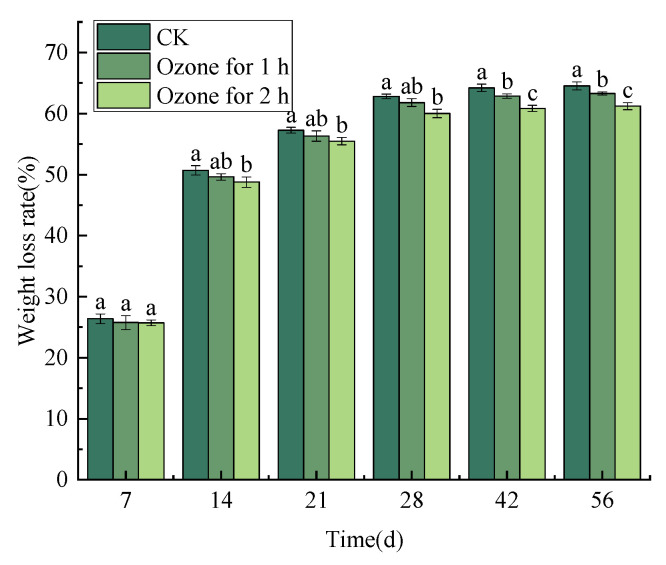
Effect of ozone treatment on weight loss rate of *C. pilosula* inoculated with *P. expansum*. Bars indicate standard error. Different letters indicate significant differences (*p* < 0.05).

Ozone fumigation treatment alleviated the weight loss of *C. pilosula* to some extent. The weight loss rate of *C. pilosula* in the control group and the ozone treatment group increased with the prolongation of storage time; especially during days 0–14 of storage, the weight loss rate of *C. pilosula* increased rapidly. Nevertheless, the weight loss rate increased slowly from the 14th to the 28th day after treatment. From the 28th day to the end of storage, the weight loss rate increase tended to be gentle. The weight loss rate in the control group was 62.81% on the 28th day of storage, while in the ozone treatment group it was 61.79% (1 h) and 60.00% (2 h), respectively. At the end of storage, the weight loss rate in the control group reached 64.51%, while the weight loss rate in the ozone treatment group was 63.28% (1 h) and 61.20% (2 h), respectively (Figure 2).

### 3.3. Effect of Ozone Treatment on Patulin Accumulation in C. pilosula Inoculated with P. expansum

**Figure 3:**
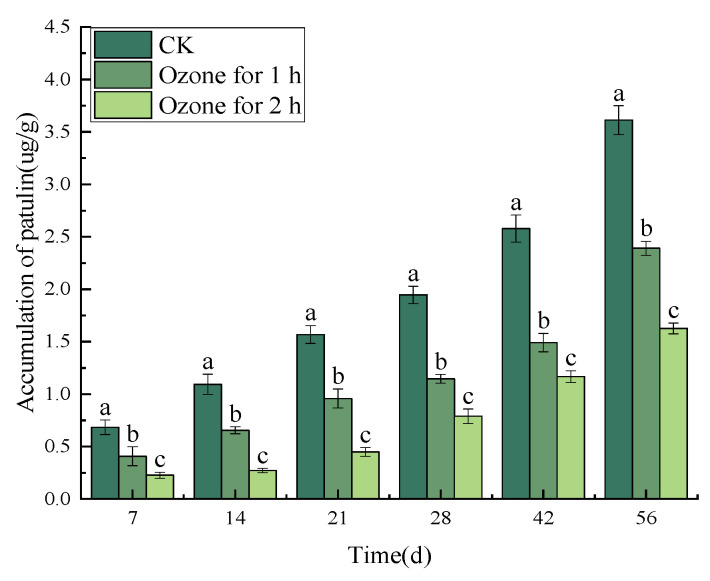
Effect of ozone treatment on patulin accumulation in *C. pilosulain* inoculated with *P. expansum*. Bars indicate standard error. Different letters indicate significant differences (*p* < 0.05).

Ozone treatment significantly (*p* < 0.05) reduced the accumulation of patulin in *C. pilosula* inoculated with *P. expansum*. The infection of *P. expansum* led to the accumulation of patulin (PAT) in the tissue of the bule mold of *C. pilosula*. With the increase in storage time, the content of PAT in the tissue of *C. pilosula* increased continuously, and the increasing trend in the control group was significantly greater than that in the ozone treatment group; the longer the ozone treatment time, the more obvious the inhibitory effect that was found. For example, on the 56th day of inoculation with *P. expansum*, the PAT content of the control group was 3.61 µg/g, while the PAT content of the ozone treatment 1 h and 2 h groups were 2.39 µg/g and 1.63 µg/g, respectively, and the PAT toxin content decreased by 33.06% and 54.85%, respectively (Figure 3).

### 3.4. Ozone Treatment Maintained the Content of Main Active Ingredients in C. pilosula Inoculated with P. expansum

**Figure 4:**
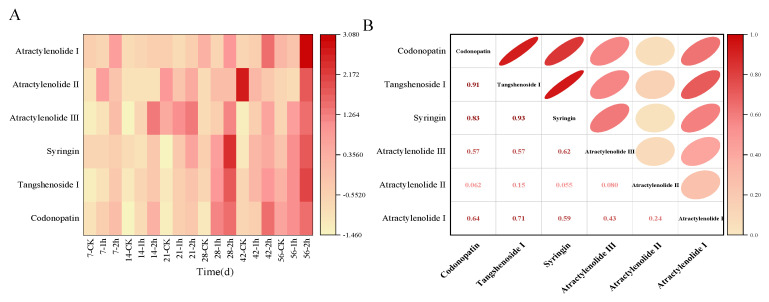
Effect of ozone treatment on the content of the main active ingredients of the blue mold of *C. pilosula*. (**A**) Thermography; (**B**) Analysis of relationship.

Ozone application markedly inhibited the *P. expansum* infection and effectively maintained the main active ingredients in *C. pilosula*. In the present study, the six main active ingredients of codonopatin, tangshenoside I, syringin, atractylenolide III, atractylenolide I, and atractylenolide II were detected in the tissue of *C. pilosula* by HPLC (Supplementary Materials, Figure S1). The change of contents for six active ingredients in *C. pilosula* inoculated with *P. expansum* in ozone treatment group and control group were shown in Figure 4. Compared with the control group, the four main active ingredients of codonopatin, tangshenoside I, syringin, atractylenolide III in *C. pilosula* after the ozone treatment maintained a good level (the darker the color, the higher the relative content of the ingredient was observed); however, the two active ingredients of atractylenolide I and atractylenolide II almost could not be detected after ozone treatment. In addition, as shown in Figure 4B, the correlation analysis presented that codonopatin was significantly positively correlated with tangshenoside I, syringin, atractylenolide III, and atractylenolide I; there was a significant positive correlation between tangshenoside I and syringin, atractylenolide III, and atractylenolide I. Syringin was significantly positively correlated with atractylenolide III (the darker the color, the stronger the correlation).

**Figure 5:**
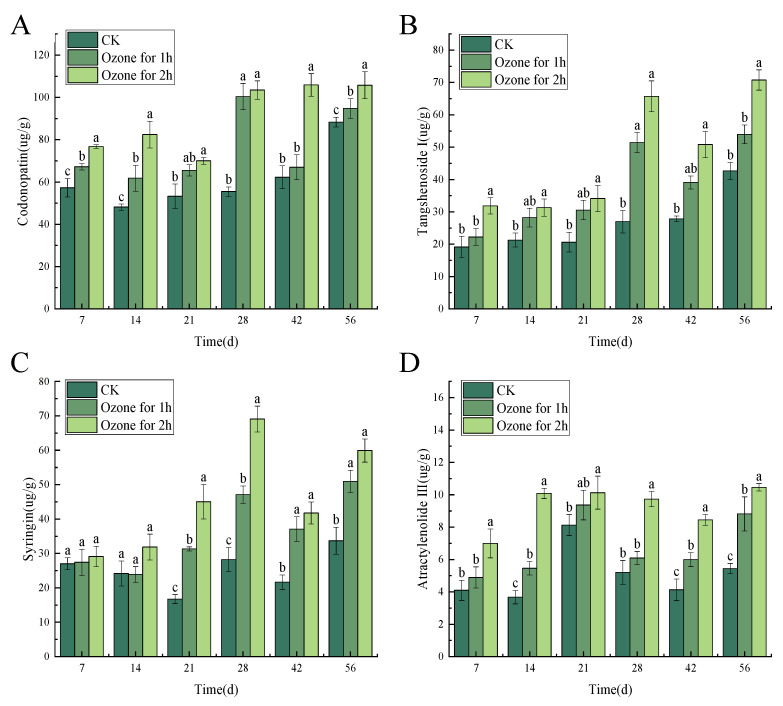
Effect of ozone treatment on the content of four main active ingredients of the blue mold of *C. pilosula*. Bars indicate standard error. Different letters indicate significant differences (*p* < 0.05). (**A**) lobetyolin; (**B**) lobetyolin I; (**C**) syringin; (**D**) atractylenolide III.

After different concentrations of ozone treatment, the content of the main active ingredients of codonopatin, tangshenoside I syringin and atractylenolide III (atractylenolide I and atractylenolide II was too low to be detected) was maintained in the *C. pilosula* tissue (Figure 5). In general, the infection of *P. expansum* led to a reduction in the content of lobetyolin, lobetyolin I, syringin and atractylenolide III. However, ozone treatment inhibited the growth of *P. expansum*, and maintained the content of codonopatin, tangshenoside I, syringin and atractylenolide III to a certain extent.

**Figure 6:**
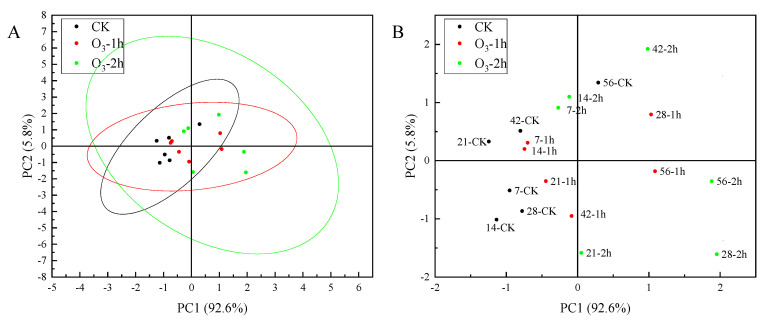
PCA score scatter plot of *C. pilosula* samples. (**A**). Load chart. (**B**). Score chart.

The principal component analysis of the main active ingredients of *C. pilosula* at different storage periods showed that the characteristic values of codonopatin, tangshenoside I and syringin were 3.79, 1.03 and 0.58, respectively. The cumulative contribution rate of codonopatin, tangshenoside I and syringin reached 89.83%. The codonopatin, tangshenoside I and syringin can be used as the evaluation indexes of the main active ingredients of *C. pilosula* at different storage times. According to the results of principal component analysis, the principal component score coefficient matrix was obtained. Taking the variance contribution rate of the top three principal components of codonopatin, tangshenoside I and syringin as the weight, the principal component comprehensive score was calculated: comprehensive score (H) = (63.08707 × F1 + 17.12968 × F2 + 9.61508 × F3)/89.83183. According to the comprehensive score of principal components, we can know the ranking (Supplementary Materials, Table S3). Combined with the PCA score diagram of the principal components in Figure 6, the score of the ozone treatment 2 h group on the 56th day was the highest, and the score of the control group on the 14th day was the lowest. The comprehensive evaluation and distribution results of the main active components of *C. pilosula* were highest in the ozone treatment 2 h group, followed by the ozone treatment 1 h group, and the control group had the lowest comprehensive score. The higher the score, the better the retention effect of the active components of *C. pilosula*. It can be seen from the results of the score that the active components of *C. pilosula* after ozone treatment were significantly higher than those of the control group.

### 3.5. Ozone Treatment Activated ROS Metabolism in C. pilosula Inoculated with P. expansum

#### 3.5.1. Ozone Treatment Suppressed the Accumulation of O_2_^−.^ and H_2_O_2_ in the Inoculated *C. pilosula*

**Figure 7:**
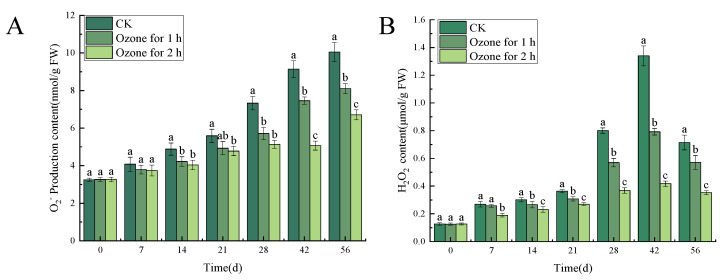
Effects of ozone treatment on O_2_^−.^ production content (**A**) and H_2_O_2_ content (**B**) in *C. pilosula*. Bars indicate standard error. Different letters indicate significant differences (*p* < 0.05).

Compared with the untreated control group, the ozone treatment significantly (*p* < 0.05) reduced the contents of O_2_^−.^ and H_2_O_2_ in *C. pilosula* inoculated with *P. expansum*. The analysis of the O_2_^−.^ production content of the inoculated *P. expansum* showed that there was a significant difference between the control group and the ozone treatment group (*p* < 0.05). However, the content of O_2_^−.^ generally increased with the increase of storage time and reached a peak on the 56th day of storage. The ozone treatment for 1 h and 2 h decreased by 19.40% and 33.33%, respectively, compared with the control group (Figure 7A). The content of H_2_O_2_ increased first, then decreased, and reached a peak on the 42nd day. After 1 h and 2 h of ozone treatment, the content of H_2_O_2_ in *C. pilosula* decreased by 41.04% and 68.66%, respectively, compared with the control (Figure 7B).

#### 3.5.2. Ozone Treatment Increased the Activities of NOX, SOD, CAT and POD in *C. pilosula*

**Figure 8:**
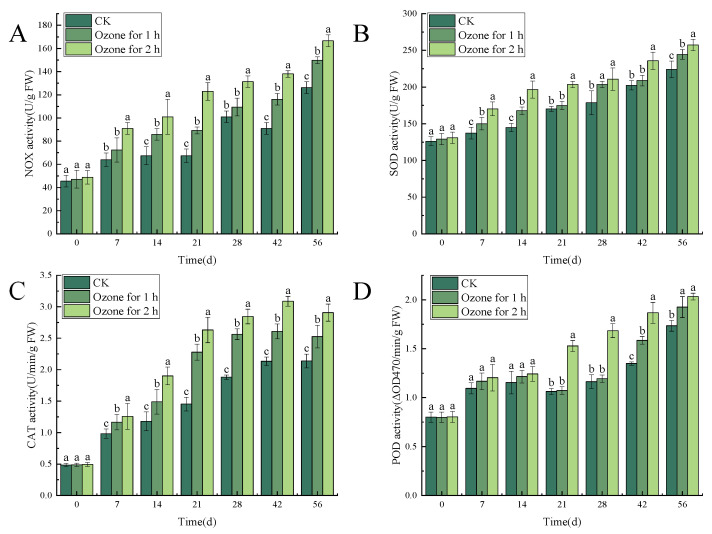
The effect of ozone treatment on the activities of NOX (**A**), SOD (**B**), CAT (**C**) and POD (**D**) in *C. pilosula*. Bars indicate standard error. Different letters indicate significant differences (*p* < 0.05).

The result showed that ozone treatment significantly (*p* < 0.05) increased the activities of NOX, SOD, CAT and POD. There were significant differences between ozone-treated and control groups in NOX activity in *C. pilosula* during different times after inoculation with *P. expansum*. NOX activity generally increased with the prolongation of storage time. The NOX activity in the ozone treatment 2 h group reached a peak of 126.25 U/g FW on the 56th day of storage, which was significantly higher than that in the control group (24.24%) and the ozone treatment 1 h group (10.10%), and NOX activity in the ozone treatment 1 h group was significantly higher than that in the control group (15.73%) (Figure 8A). Similar results were observed in the activities of SOD, CAT and POD. SOD activity gradually increased with the extension of storage time and was markedly higher in the ozone treatment 2 h group than in the control group (12.91%) and the 1 h treatment group (4.99%), respectively, on the 56th day (Figure 8B). CAT activity in the ozone treatment group reached a peak on the 42nd day of storage, and activity in the ozone treatment 2 h group was significantly higher than in the control group (26.46%) and ozone treatment 1 h group (13.06%) (Figure 8C). A similar changing trend was found in POD activity (Figure 8D).

### 3.6. Effects of Ozone Treatment on Cell Membrane Permeability and MDA Content in C. pilosula

**Figure 9:**
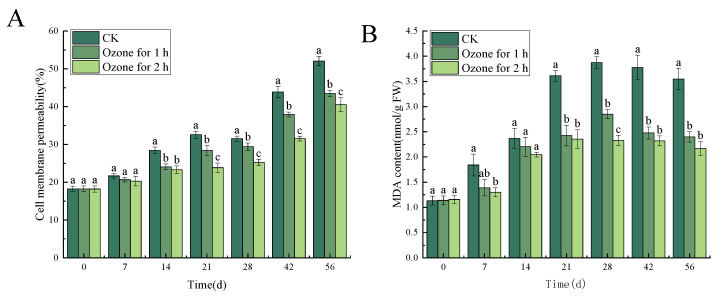
Effects of ozone treatment on cell membrane permeability (**A**) and MDA content (**B**) in *C. pilosula*. Bars indicate standard error. Different letters indicate significant differences (*p* < 0.05).

Compared with the untreated control group, the ozone treatment significantly (*p* < 0.05) reduced cell membrane permeability and MDA content of *C. pilosula* tissue. Cell membrane permeability and MDA content are employed to evaluate the cell membrane integrity of plant tissues. The cell membrane permeability in ozone-treated *C. pilosula* inoculated with *P. expansum* was significantly lower than it was in the control group. Similarly, the MDA content was also significantly lower than it was in the control group (*p* < 0.05), and the cell membrane permeability and MDA content decreased with the prolongation of ozone treatment time. After 56 days of inoculation, the cell membrane permeability in ozone-treated groups for 1 h and 2 h, respectively, decreased by 16.57% and 22.15%, when compared with the control group (Figure 9A). The MDA content in ozone-treated groups for 1 h and 2 h decreased by 32.35% and 38.87%, respectively, compared with the control group (Figure 9B).

## 4. Discussion

### 4.1. Ozone Treatment Inhibits the Occurrence of the Blue Mold of C. pilosula

Our previous studies suggested that *P. expansum* is the main pathogen causing fresh *C. pilosula* blue mold during the postharvest storage period, which seriously impairs the quality of the fresh *C. pilosula* after harvest [19]. In this study, it was found that 2 mg L^−1^ ozone fumigation treatment significantly reduced the disease incidence of the blue mold of *C. pilosula*, inhibited the development of the blue mold of *C. pilosula*, reduced the accumulation of patulin, and alleviated the weight loss of *C. pilosula* to some extent. The longer the ozone treatment time, the more significant the effect that was observed. The PAT content in the ozone treatment for 2 h group was 1.63 µg/g on the 56th day of inoculation with *P. expansum*; compared with the control group, the PAT content decreased by 54.85% and the accumulation of toxins was greatly reduced but it still exceeded the maximum tolerable daily intake of 0.4 µg/kg stipulated by the Joint Expert Committee on Food Additives (JECFA) of the World Health Organization [25]. Luo et al. [15] also indicated that gaseous ozone application significantly reduced the disease incidence of postharvest kiwifruit, maintained high fruit hardness and prolonged fruit shelf life. Similarly, Li et al. [10] suggested that ozone treatment not only significantly inhibited the growth of potato dry rot caused by *F. sulphureum*, but also controlled diacetoxyscirpenol accumulation. Smila et al. [26] found that ozone water (1.5 mg L^−1^ for 2 min) treatment directly acted on *P. expansum*, *P. digitatum*, *P. italicum*, *Botrytis cinerea* and *Rhizopus stolonifer*, and the killing rate of the above spores was 95–100%, which showed that ozone treatment can reduce the degree of pathogen infection to the host, suppress the disease incidence, and, ultimately, control the occurrence of postharvest diseases by inhibiting the growth of pathogenic fungi. In fact, in addition to the direct inhibitory effect, ozone treatment also can stimulate ROS metabolism and induce hosts’ resistance, and, finally, control the disease development. It has been shown that appropriate concentration of ozone treatment can effectively delay the browning of mushrooms by increasing the activities of POD, SOD and CAT, maintain a high level of total phenols and soluble protein content, and reduce the total number of colonies, molds and yeasts in mushrooms [27].

### 4.2. Effect of Ozone Treatment on the Content of Main Effective Components in C. pilosula Inoculated with P. expansum

**Figure 10:**
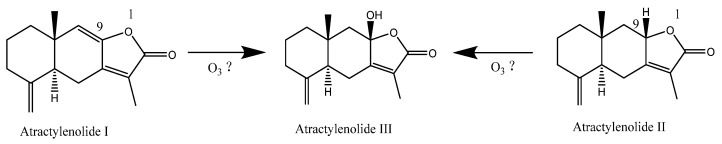
Schematic diagram of active ingredient transformation of *C. pilosula*.

As we know, the medicinal value of *C. pilosula* is mainly reflected in its main active ingredients. The main active ingredients of *C. pilosula* are codonopatin, tangshenoside I, syringin, atractylenolide III, atractylenolide I, and atractylenolide II, which can enhance immunity, microcirculation and hematopoietic function, and have obvious improvement effects on diabetes, liver protection and base repair. In this study, *P. expansum* infection, while impairing the quality of *C. pilosula* and producing patulin, significantly influenced these active ingredients; in particular, *P. expansum* infection significantly reduced the content of codonopsis glycosides, which may be related to the consumption of codonopsis glycosides in *C. pilosula* tissue due to fungal growth and metabolism [28]. Xi et al. [29] found that ozone treatment effectively reduced the infection of pathogenic fungus to *Angelica sinensis*, inhibited the development of disease and reduced the accumulation of mycotoxins. Hu et al. [30] also found that the content of flavonoid glycosides in *Radix Astragali* was significantly reduced after *Aspergillus flavus* infection. Ozone application markedly inhibited *P. expansum* infection and effectively maintained the main active ingredients in *C. pilosula*. For instance, the content of codonopatin, tangshenoside I, syringin and atractylenolide III were well maintained, and the content of atractylenolide III in particular significantly increased; however, the contents of atractylenolide I and atractylenolide II decreased. We speculate that the active ingredients of atractylenolide I and atractylenolide II convert into atractylenolide III under the action of ozone. For instance, under the action of ozone, the hydrogen on the ninth double bond of atractylenolide I and the ninth carbon atom of atractylenolide II undergoes a series of chemical reactions to form hydroxyl groups, thereby obtaining atractylenolide III (Figure 10).

### 4.3. Effects of Ozone Treatment on ROS Metabolism in C. pilosula Tissue

Reactive oxygen species (such as O_2_^−.^ and H_2_O_2_) is the by-product during the interaction between plant and pathogen, and ROS at the appropriate concentration acts as signal molecules to mediate downstream signaling pathways and regulate the host’s defense response [31]. However, high concentrations of ROS will attack the host’s plasma membrane structure and cause lipid peroxidation, which ultimately leads to cell membrane damage and pathogen invasion, eventually leading to disease occurrence. In the present study, the ozone treatment significantly reduced the contents of O_2_^−.^ and H_2_O_2_ in *C. pilosula* inoculated with *P. expansum*, when compared with the untreated control group. The reason may be that, when *P. expansum* infected *C. pilosula*, the tissue of *C. pilosula* suffered biological stress, which resulted in the increase of the content of O_2_^−.^ and H_2_O_2_. When the content of O_2_^−.^ and H_2_O_2_ exceeds the range of the oxidative defense of *C. pilosula*, the lipid peroxidation of *C. pilosula* cell membrane leads to oxidative damage, reduces cell activity, accelerates the invasion of pathogen, and is conducive to the decay and deterioration of *C. pilosula*. However, ozone application, on the one hand, inhibits the growth of pathogens, and once the growth of pathogens is inhibited, the development of disease will be controlled. More importantly, ozone treatment stimulates ROS metabolism in plant tissue and maintains oxidative homeostasis. It is well known that NOX and SOD are the main enzymes that produce O_2_^−.^ and H_2_O_2_. NOX mainly regulates the production of O_2_^−.^, and SOD catalyzes the disproportionation reaction of O_2_^−.^ to produce H_2_O_2_ and O_2_. CAT and POD further decompose H_2_O_2_ into H_2_O and O_2_, thus protecting cells from ROS damage. In this study, the activities of NOX, SOD, CAT and POD increased with the prolongation of storage time, and in the ozone treatment group were significantly higher than in the control group, which promoted the antioxidant system and maintained the steady state of *C. pilosula* tissue cells. This result is consistent with Lin et al. [32], who found that ozone treatment significantly increased the activities of SOD, CAT and POD, improved the resistance against disease, maintained postharvest quality and prolonged shelf life of postharvest *Toona sinensis*. Therefore, we infer that, although ozone stimulates the accumulation of ROS, at the same time, ozone application increased the activities of the antioxidant enzymes, which is attributed to eliminating or removing the excessive ROS, to avoid the membrane lipid peroxidation due to ROS excessive accumulation.

As we expected, ozone treatment reduced cell membrane permeability and MDA content of *C. pilosula* tissue. The result agreed with the report by Liang et al. [33], who found that ozone treatment maintained the hardness of postharvest tomatoes by reducing cell membrane permeability and inhibiting the accumulation of MDA, thereby prolonged the shelf life of tomatoes. Similarly, Wang et al. [34] also suggested that fresh-cut kiwifruit treated with 1 mg/L ozone for 10 min significantly inhibited the activity of cell wall polysaccharide degrading enzymes, reduced MDA content, maintained protopectin and cellulose levels, and delayed fruit softening and decay.

## 5. Conclusions

Ozone treatment at a concentration of 2 mg L^−1^ for 2 h significantly reduced the *P. expansum* infection, alleviated the weight loss of *C. pilosula*, and suppressed the accumulation of patulin. Ozone treatment maintained the contents of the main active ingredients of lobetyolin, lobetyolin I, syringin and atractylenolide III. The mechanism is attributed to ozone treatment stimulating ROS metabolism of *C. pilosula* tissues, enhanced the activity of SOD, NOX, POD and CAT, which avoided the oxidative stress caused by the excessive accumulation of O_2_^−.^ and H_2_O_2_ in the tissues, better maintained the integrity of the cell membrane, consequently improved the resistance of *C. pilosula* against *P. expansum*, and inhibited the occurrence of postharvest blue mold and maintained the main active ingredients of *C. pilosula*. This study provides a theoretical basis for ozone treatment to inhibit the growth of *P. expansum* and control postharvest diseases of Chinese herbal medicine. In follow-up studies, the molecular mechanism of ozone treatment to suppress the growth of *P. expansum*, inhibit the accumulation of patulin and manage the blue mold of *C. pilosula* needs to be further studied.

## Data Availability

The data presented in this study are included in the article; further inquiries can be directed to the corresponding author.

## Supplementary Materials

The following supporting information can be downloaded at: https://www.mdpi.com/article/10.3390/jof10030163/s1, Figure S1: Chromatograms separation by HPLC for the main active ingredients in *C. pilosula*.; Table S1: Disease classification standard; Table S2: Mobile phase gradient elution procedure; Table S3: Principal component eigenvalues, variance contribution rate and cumulative variance contribution rate.
