# Supplementary material for: Ozone Application Suppressed the Blue Mold Development and Maintained the Main Active Ingredients Content of Postharvest Fresh Codonopsis pilosula during Storage
Source: J Fungi (Basel). 2024 Feb 20;10(3):163. doi: 10.3390/jof10030163 (PMC10971366; doi:10.3390/jof10030163)
Supplement: Supplementary file 1 [file jof-10-00163-s001.zip › jof-2809244-supplementary.pdf]

Supplementary Materials:

Supporting Table S1. Disease classification standard.

[a] The percentage of diseased area to the total area of *C. pilosula*.

Disease classification	Symptoms described[a]	Pathogenicity
0	0	nonpathogenic
1	1% -5%	low pathogenicity
3	6% - 25%	low pathogenicity
5	26% -50%	neutral pathogenicity
7	51% -75%	neutral pathogenicity
9	75% -100%	highly pathogenic

Supporting Table S2. Mobile phase gradient elution procedure.

Unit	Information	
Apparatus	Agilent 1260 high performance liquid chromatograph	
Chromatographic column	Symmetry C-18 (250 nm×4.6 nm×5 μm)	
Detector	UV detector	
Detection wavelength	220 nm and 276 nm	
Column temperature	30 °C	
Injection volume	20 μL	
Flow rate	1.0 mL • min^{-1}	
Test condition	The mobile phase A: Acetonitrile<br>The mobile phase B: 0.1 % Phosphoric acid aqueous solution	Isocratic elution<br>0min：10% Acetonitrile（A）<br>0-5min：25% Acetonitrile（A）<br>5-8min：45% Acetonitrile（A）<br>8-15min：75% Acetonitrile（A）<br>15-22min：85% Acetonitrile（A）

Supporting Table S3. Principal component eigenvalues, variance contribution rate and cumulative variance contribution rate.

Principal component	Eigenvalue	Variance contribution rate (%)	accumulated variance contribution rate (%)
codonopatin	3.654529	60.7549	60.7549
tangshenoside I	1.53725	25.62083	86.37572
syringin	0.44712	7.95202	94.32774
atractylenolide III	0.1528	2.54661	96.87435
atractylenolide II	0.10926	1.82095	98.6953
atractylenolide I	0.07828	1.3047	100

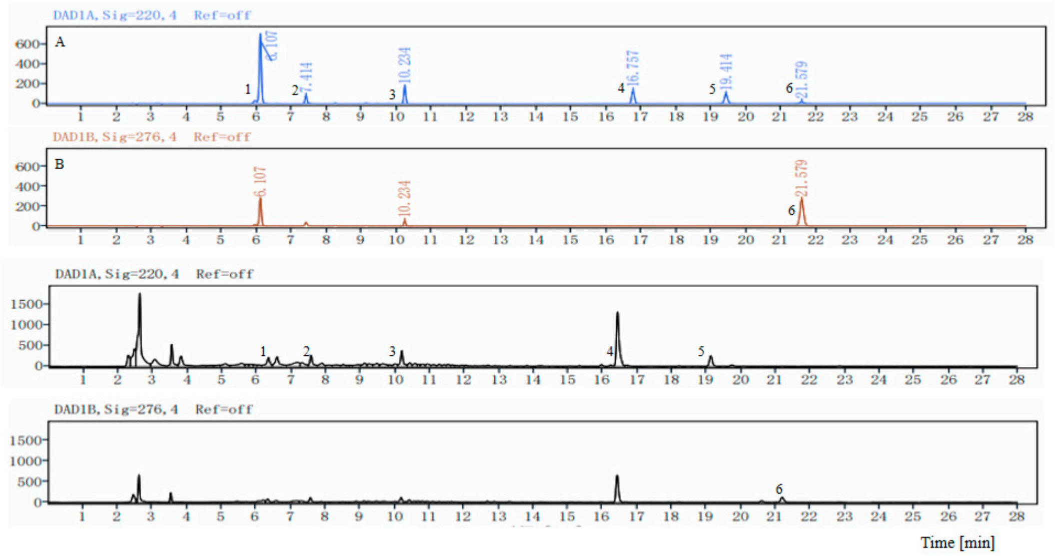

Supporting Figure S1. Chromatograms separation by HPLC for the main active ingredients in *C. pilosula*. A. Standard sample (220 nm); B. Standard sample (276 nm); C. sample of *Codonopsis pilosula* (220 nm); D. sample of *Codonopsis pilosula* (276 nm). 1: codonopatin; 2: tangshenoside I; 3: syringin; 4: atractylenolide III; 5: atractylenolide II; 6: atractylenolide I.
